# Overexpression of *OsRbohH* Enhances Heat and Drought Tolerance through ROS Homeostasis and ABA Mediated Pathways in Rice (*Oryza sativa* L.)

**DOI:** 10.3390/plants13172494

**Published:** 2024-09-05

**Authors:** Yating Chen, Rui Zhang, Rujie Wang, Jiangdi Li, Bin Wu, Haiwen Zhang, Guiqing Xiao

**Affiliations:** 1 College of Bioscience and Biotechnology, Hunan Agricultural University, Changsha 410128, China; chenyt9725@163.com (Y.C.); sz20220671@stu.hunau.edu.cn (R.Z.); wangrj0217@163.com (R.W.); jdli1998@163.com (J.L.); 18873109495@163.com (B.W.); 2 Biotechnology Research Institute, Chinese Academy of Agricultural Sciences, Beijing 100081, China; zhanghaiwen@caas.cn

**Keywords:** rice, *OsRbohH*, heat tolerance, drought stress, ROS, ABA

## Abstract

Respiratory burst oxidase homologs (Rbohs) are the primary producers of reactive oxygen species (ROS), which have been demonstrated to play critical roles in plant responses to abiotic stress. Here, we explored the function of *OsRbohH* in heat and drought stress tolerance by generating overexpression lines (*OsRbohH*-OE). *OsRbohH* was highly induced by various abiotic stress and hormone treatments. Compared to wild-type (WT) controls, *OsRbohH*-OE plants exhibited enhanced tolerance to heat and drought, as determined by survival rate analyses and total chlorophyll content. Histochemical staining revealed that *OsRbohH*-OE accumulated less ROS. This is consistent with the observed increase in catalase (CAT) and peroxidase (POD) activities, as well as a reduced electrolyte leakage rate and malondialdehyde (MDA) content. Moreover, *OsRbohH*-OE exhibited enhanced sensitivity to exogenous abscisic acid (ABA), accompanied by altered expression levels of ABA synthesis and catabolic genes. Further analysis indicated that transgenic lines had lower transcripts of ABA signaling-related genes (*OsDREB2A*, *OsLEA3*, *OsbZIP66,* and *OsbZIP72*) under heat but higher levels under drought than WT. In conclusion, these results suggest that *OsRbohH* is a positive regulator of heat and drought tolerance in rice, which is probably performed through OsRbohH-mediated ROS homeostasis and ABA signaling.

## 1. Introduction

To cope with extreme weather, such as high temperatures, freezing conditions, and water deficiency, plants generate multiple signaling molecules, including ROS, phytohormones, nitric oxide (NO), calcium ions (Ca^2+^), and others, to maintain cellular homeostasis and minimize damage [1,2,3]. ROS plays a crucial role in activating stress–defense responses at low concentrations as a critical second messenger; upon reaching a certain threshold, it triggers oxidative damage, ultimately leading to cell death [4,5]. Thus, it is crucial to balance ROS generation and scavenging under stress conditions [6]. Phytohormones are another central regulator of stress tolerance in plants [7]. Increasing evidence has shown that abscisic acid (ABA) regulates plant water balance and tolerance in response to heat and drought. For instance, in *Arabidopsis thaliana*, *ZmWRKY106*, *HSFA6b*, *SlHsfA3*, and *AtUNC-93* have been demonstrated to improve heat tolerance by upregulating stress-related genes involved in the ABA-signaling pathway [8,9,10,11]. *OsOLP1*, *OsWRKY97*, and *OsAAI1* promote ABA accumulation and enhance drought resistance in rice [12,13,14].

Rbohs, also known as Nicotinamide adenine dinucleotide phosphate (NADPH) oxidases, are crucial enzymes in generating ROS. Previous research has indicated that Rbohs are integral plasma membrane proteins that contain six transmembrane domains, an N-terminal region with two Ca^2+^-binding EF-hands, and a C-terminal FAD-binding domain [15,16]. Rbohs proteins are capable of transferring electrons from the cytosolic donor NADPH to the extracellular receptor O_2_ through FAD and two hemes, producing O_2_^−^ and subsequently undergoing dismutation to generate H_2_O_2_ [17], which is more stable than O_2_^−^ [18]. Up to now, over 150 *Rboh* genes have been identified in the plant kingdom [19], including in tomato (*Solanum Lycopersicum*) [20], tobacco (*Nicotiana tabacum*) [21], potato (*Solanum tuberosum*) [22], barrel medic (*Medicago truncatula*) [23], rice [24], maize (*Zea mays*) [25], and *Arabidopsis* [18]. For instance, the tomato Rboh family contains 11 members [26]. *SlRbohB* positively regulates tolerance to drought stress [27], while silencing *SlRBOH1* compromises plant thermotolerance [28]. In *Arabidopsis,* there are ten genes encoding NADPH oxidase [29]. Five *AtRboh* genes (e.g., *AtRbohB*, *AtRbohC*, *AtRbohE*, *AtRbohJ*, *AtRbohH*) are involved in plant growth and development [30,31,32,33]. Moreover, at least three *AtRboh* genes (e.g., *AtRbohD*, *AtRbohF*, *AtRbohI*) play crucial roles in response to adversity [34,35].

In rice, nine *Rboh* members have been reported to date, and they widely participate in responses to various stressors [36]. For instance, *OsRbohA*, *OsRbohB*, *OsRbohC*, *OsRbohE*, *OsRbohF*, *OsRbohH*, and *OsRbohI* are all involved in ROS-dependent immunity against fungal diseases [37]. Knockout of *OsRbohA* reduced pollen viability and attenuated tolerance to salt and drought, which was associated with decreased intracellular ROS levels [38,39]. Overexpression of *OsRbohB* enhances the capability of drought tolerance in rice [40]. *OsRbohB* and *OsRbohE* are vital in regulating antioxidant stress [17]. *OsRbohD* and *OsRbohH* act as negative regulators of the response to saline–alkaline stress in plants [41]. Although the function of these *OsRboh* genes has been well studied in many respects, whether they are involved in regulating the response to extreme temperature is largely unclear.

Here, we dissected *OsRbohH* expression profiles and performed a detailed functional analysis of *OsRbohH* in abiotic stress tolerance by overexpressing *OsRbohH* in transgenic rice. Our results revealed that *OsRbohH*-OE plants confer improved heat tolerance and drought resistance, characterized by reduced chlorophyll degradation, decreased ROS accumulation, attenuated membrane lipid peroxidation, and enhanced antioxidant enzyme activity. Further studies showed increased sensitivity to ABA in *OsRbohH*-OE plants, accompanied by altered expression of ABA synthesis and degradation genes. These results suggest that *OsRbohH* is a positive regulator of heat and drought stress in rice, which might be mediated by ROS homeostasis and ABA signaling.

## 2. Results

### 2.1. Expression Patterns of OsRbohH

**Figure 1:**
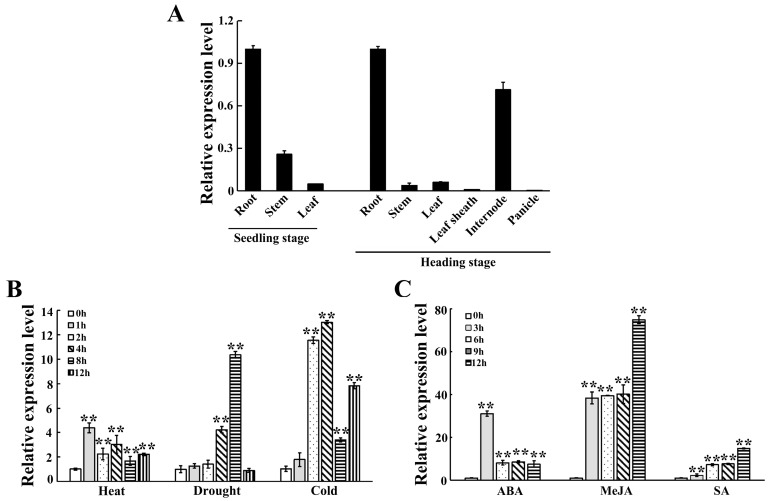
Expression profiles of *OsRbohH.* (**A**) Detection of *OsRbohH* expression in different tissues and growth stages using qRT-PCR. (**B**) Expression patterns of *OsRbohH* under heat (42 °C), simulated drought (20% PEG6000), and cold (4 °C). (**C**) Expression patterns of *OsRbohH* responses to ABA (400 µM), MeJA (100 µM), and SA (100 µM). The expression levels are represented as fold changes relative to those in roots or at 0 h. **: *p* ≤ 0.01.

To determine the expression profiles of the *OsRbohH* gene, we conducted quantitative real-time PCR (qRT-PCR) to analyze the transcript levels of *OsRbohH* in different tissues and organs of rice. At the seedling stage, the expression of *OsRbohH* was notably high in roots, whereas it was low in leaves. During the heading stage, *OsRbohH* transcripts were abundant in roots but minimal in panicles (Figure 1A). The RPKM value analysis also demonstrated that *OsRbohH* was relatively highly expressed in roots and undetectable in leaves (Table S1). These results indicate that *OsRbohH* expression is tissue specific.

Next, we analyzed the effects of abiotic stressors and phytohormones on the *OsRbohH* transcripts. The mRNA levels of *OsRbohH* were substantially stimulated by heat, drought, and cold stress (Figure 1B). In detail, *OsRbohH* responded rapidly during the early stages of heat stress. Conversely, under drought and cold conditions, the expression of *OsRbohH* gradually increased during the later stages of stress. Meanwhile, the transcriptional expression of the gene was also induced by the application of exogenous ABA, MeJA (methyl jasmonate), and SA (salicylic acid) (Figure 1C). In ABA treatment, the mRNA level of *OsRbohH* was significantly elevated by nearly 30 times at 3 h and then decreased gradually. With the MeJA and SA treatments, the transcripts of *OsRbohH* continued to rise within 12 h. These findings suggest that *OsRbohH* has different modes for responding to various stressors.

### 2.2. Overexpression of OsRbohH Enhanced Heat Stress Tolerance

To further verify whether *OsRbohH* was involved in heat tolerance in rice, we generated *OsRbohH*-overexpressing transgenic plants (*OsRbohH*-OE) in the Nipponbare (WT) background. Two independent lines (*OsRbohH*-OE1 and *OsRbohH*-OE2) accumulated *OsRbohH* transcripts at a >3-fold higher level compared with the parental line (Figure S1).

**Figure 2:**
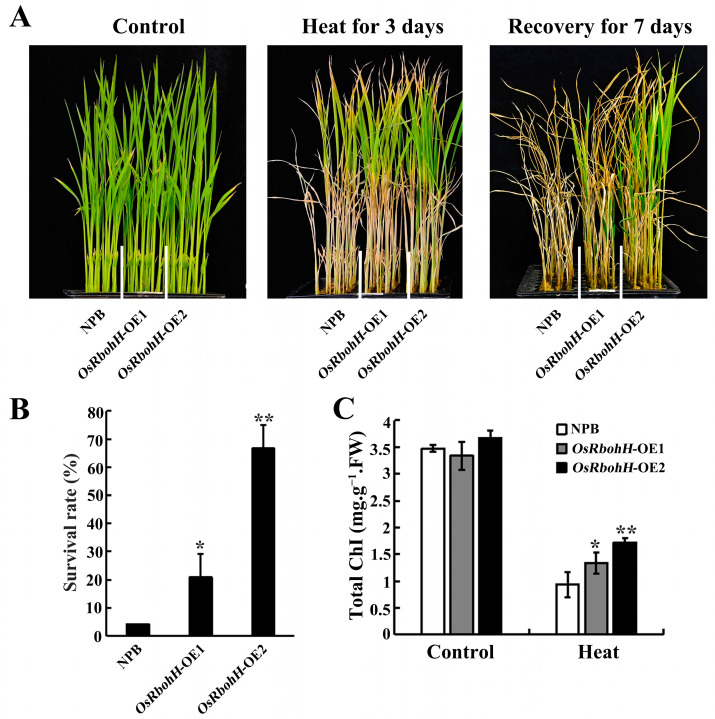
Heat phenotypes of *OsRbohH*-OE plants (**A**) Phenotypes of *OsRbohH*-OE and WT plants before high-temperature treatment (left panel), after 3 days of heat stress at 45 °C (middle panel), and after 1 week of recovery (right panel). (**B**) Survival rates. (**C**) Total chlorophyll content of *OsRbohH*-OE and WT plants. FW, Fresh weight. **: *p* ≤ 0.01; *: *p* ≤ 0.05.

Two-week-old seedlings were treated for 3 days at 45 °C and then recovered at 28 °C for 7 days; the *OsRbohH*-OE plants showed better phenotypic performances, whereas almost all leaves of WT plants displayed rolling and wilting (Figure 2A). The statistics rate showed that 20.83% of *OsRbohH*-OE1, 66.67% of *OsRbohH*-OE2, and 4.17% of the WT plants survived (Figure 2B). Consistent with these phenotypes, the total chlorophyll content in *OsRbohH*-OE lines under heat stress was significantly higher than in WT plants (Figure 2C). These results demonstrate that *OsRbohH* overexpression can enhance tolerance to heat stress in rice.

### 2.3. Overexpression of OsRbohH Enhanced Drought Stress Tolerance

**Figure 3:**
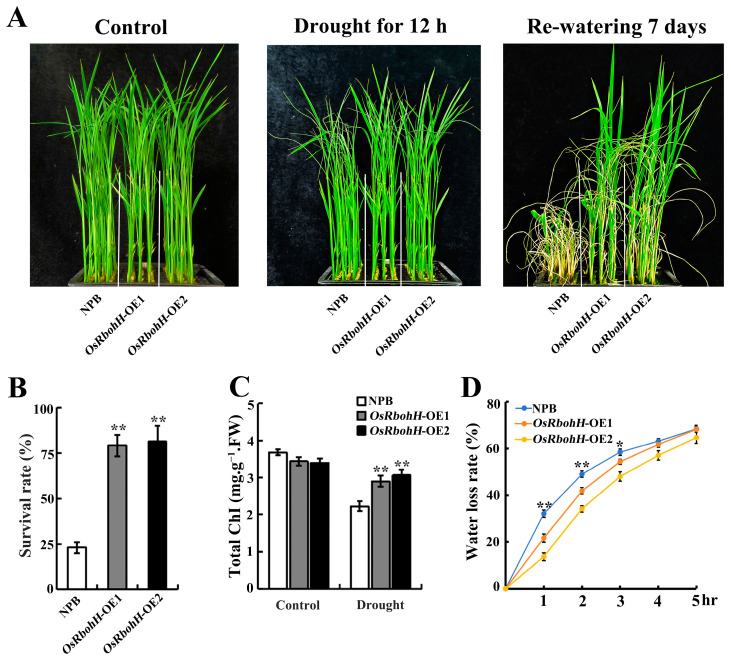
Drought phenotypes of *OsRbohH*-OE lines. (**A**) Phenotypes of the *OsRbohH*-OE and WT plants before treatment (left panel), after 12 h of drought (middle panel), and after 7 days of recovery (right panel). (**B**) Survival rates. (**C**) Total chlorophyll content of *OsRbohH*-OE and WT plants. FW, Fresh weight. (**D**) The water loss rate of detached leaves from *OsRbohH*-OE and WT plants was measured at specific time points within 5 h after detachment (n = 3, with each replicate containing 5 plants). **: *p* ≤ 0.01; *: *p* ≤ 0.05.

Frequently, high temperatures are highly correlated with drought [42]. To confirm whether *OsRbohH* was involved in the response to drought stress, two-week-old seedlings were treated with 20% PEG6000 for 12 h. The *OsRbohH*-OE plants exhibited a slightly withered phenotype compared to WT (Figure 3A). After 7 days of re-watering, approximately 80% of *OsRbohH*-OE and 30% of WT plants recovered (Figure 3B). Consistent with the drought-resistant phenotype, the total chlorophyll content of *OsRbohH*-OE plants was also significantly higher than that of WT after drought treatment (Figure 3C). Additionally, the leaves of the *OsRbohH*-OE lines lost water more slowly than those of WT, with marked differences observed at the first three treatment time points (Figure 3D). Our further findings demonstrated that *OsRbohH*-OE lines also exhibited improved phenotypes under soil drought stress conditions (Figure S2A,B), suggesting that *OsRbohH* may positively regulate drought resistance in rice.

### 2.4. OsRbohH Modulates ROS Homeostasis in Heat and Drought Stress

**Figure 4:**
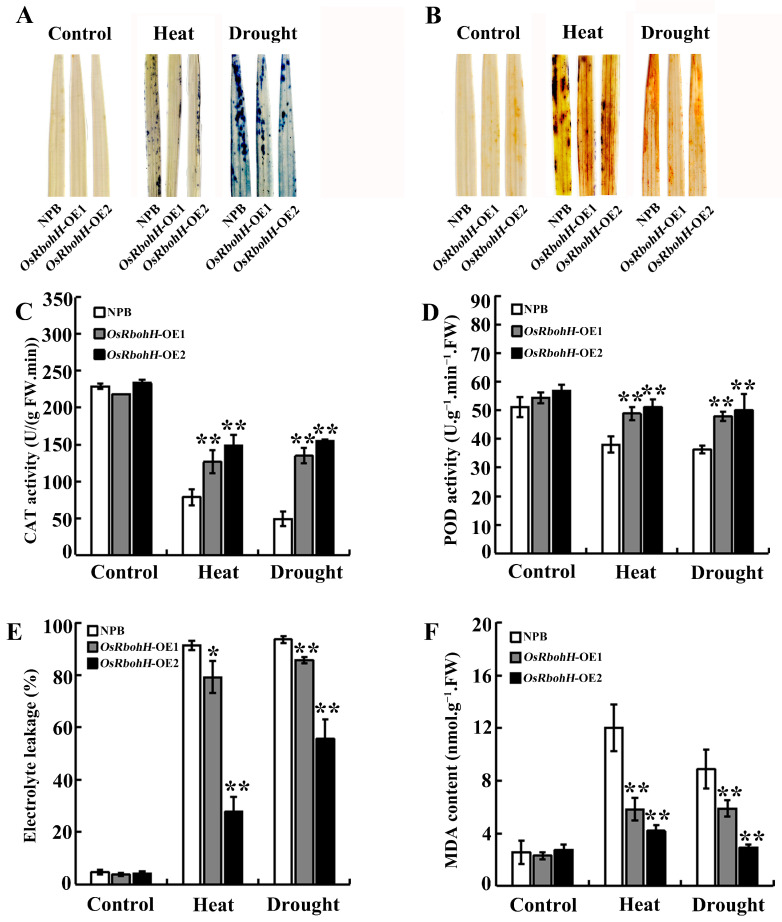
Physiological testing of *OsRbohH*-OE and WT plants under stress conditions. (**A**) NBT staining. (**B**) DAB staining. (**C**) CAT activity. (**D**) POD activity. (**E**) Relative electrolyte leakage rate. (**F**) MDA content. **: *p* ≤ 0.01; *: *p* ≤ 0.05.

ROS bursts are accompanied by adverse situations, leading to plant oxidative damage [6]. Histochemical staining was conducted to detect the levels of ROS in *OsRbohH*-OE and WT plants. The results showed that, whether it was heat or drought stress, fainter blue spots were observed in the *OsRbohH*-OE leaves compared to those in WT by nitroblue tetrazolium (NBT) staining (Figure 4A), while deeper reddish brown was observed in the WT leaves compared to *OsRbohH*-OE by 3,3′-diaminobenzidine (DAB) staining, reflecting low levels of O_2_^−^ and H_2_O_2_ accumulation in the transgenic plants, respectively (Figure 4B). Then, we turned to a quantification of the antioxidant enzyme activity. CAT and POD activity were higher in *OsRbohH*-OE than in WT plants (Figure 4C,D). Moreover, the leaves of *OsRbohH*-OE lines exhibited reduced levels of electrolyte leakage and decreased MDA content, which are essential indicators of cellular oxidative membrane damage (Figure 4E,F). These results demonstrate that *OsRbohH* transgenic plants have better ROS homeostasis maintenance when faced with adverse conditions.

### 2.5. Overexpression of OsRbohH Enhanced Sensitivity to Exogenous ABA

**Figure 5:**
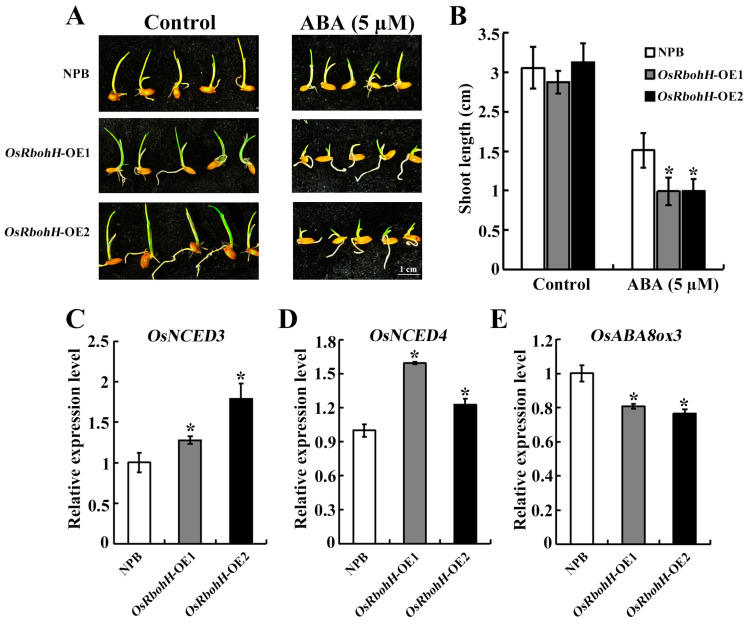
*OsRbohH* transgenic plants had enhanced sensitivity to exogenous ABA. (**A**) ABA sensitivity assay for WT and *OsRbohH*-OE plants. (**B**) Statistical data on shoot length. (**C**–**E**) Expression levels of ABA biosynthesis genes (*OsNCED3* and *OsNCED4*) and an ABA-degrading gene (*OsABA8ox3*) in *OsRbohH*-OE and WT plants. *: *p* ≤ 0.05.

*OsRbohH* was strongly induced by exogenous ABA (Figure 1C). To determine whether ABA mediated heat and drought resistance in *OsRbohH*-OE plants, we first examined the sensitivity of rice plants to exogenous ABA at the germination stage. *OsRbohH*-OE lines treated with 5 µM ABA showed a significant reduction in shoot length compared to WT (Figure 5A,B). Further analysis revealed that the expression of ABA biosynthetic genes *OsNCED3* and *OsNCED4* was upregulated (Figure 5C,D), whereas *OsABA8ox3,* which is involved in ABA degradation, was downregulated in *OsRbohH*-OE plants (Figure 5E). These results demonstrate that *OsRbohH*-OE plants display enhanced heat and drought tolerance, which might be related to their endogenous ABA content.

### 2.6. OsRbohH Is Involved in the ABA-Mediated Regulatory Pathway

**Figure 6:**
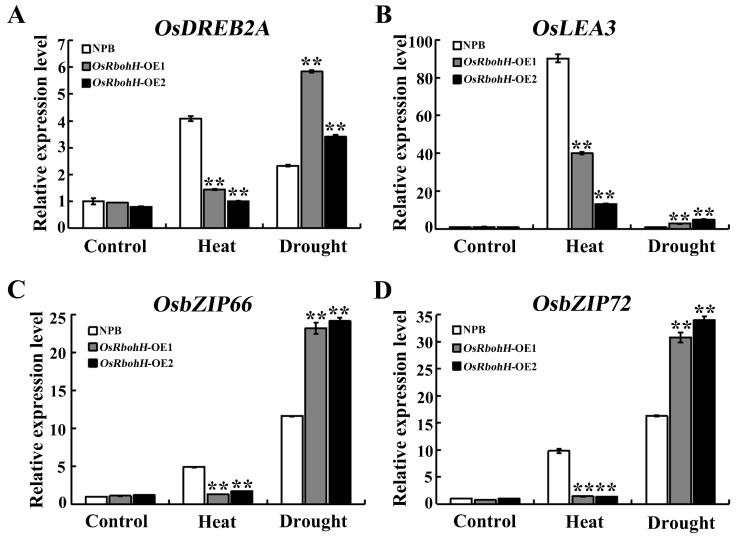
Expression analysis of ABA signaling-related genes in *OsRbohH*-OE lines. Expression analysis of (**A**) *OsDREB2A*, (**B**) *OsLEA3*, (**C**) *OsbZIP66*, and (**D**) *OsbZIP72* in *OsRbohH*-OE and WT plants under heat and drought conditions. **: *p* ≤ 0.01.

ABA is considered a crucial stress phytohormone [43]. To investigate whether *OsRbohH* was involved in the abiotic stress response through the ABA-dependent pathway, we performed RT-qPCR to analyze the expression of ABA signaling-related genes (*OsDREB2A*, *OsLEA3*, *OsbZIP66*, and *OsbZIP72*). Under non-stress conditions, there were no significant differences in the expression of these four genes between *OsRbohH*-OE and WT plants. Once the rice plants were subjected to heat or drought treatment, the transcript levels of these genes were upregulated in all transgenic lines and WT plants. The differences in response to heat tolerance showed that the expression levels of these four genes were significantly lower in *OsRbohH*-OE plants than in WT plants. Notably, *OsbZIP72* decreased about 9-fold expression in the *OsRbohH*-OE group compared to WT. However, an opposite trend was observed under drought stress. The transcript levels of these four genes were dynamically upregulated in *OsRbohH*-OE plants (Figure 6). Therefore, our findings indicate that overexpression of *OsRbohH* changes the expression level of ABA signaling-related genes to a certain extent regarding the stress response. Previous research has shown that plants promote stomatal closure by inducing ABA signal transduction [43]. Stomata are open to facilitate evaporative cooling under high-temperature stress and are closed to conserve water during drought stress [44]. In conclusion, according to these results, in the presence of heat and drought stress, we suggest that overexpression of *OsRbohH* has a positive effect on rice plants’ growth and stomata state via an opposite effect on ABA signaling-related genes, which further explains its role in promoting abiotic stress tolerance in rice.

## 3. Discussion

With global warming, high temperatures and droughts have occurred frequently worldwide. These stressors are estimated to affect over 30% of crop production [45]. Many studies have shown that the rice Rboh family is crucial in responding to unfavorable environmental conditions [37,38,39,40,41]. However, the role of these NADPH oxidases under high-temperature stress has not been reported. The finding that OsRbohH shares up to 58.12% amino acid identity with AtRbohB [46] and that *atrbohB* mutants exhibit a significantly sensitive phenotype after heat treatment [30] inspired us to investigate whether *OsRbohH* contributes to heat tolerance in rice. To confirm this hypothesis, we first analyzed the expression patterns of *OsRbohH.* Our findings indicate that *OsRbohH* expression was significantly upregulated in response to heat stress (Figure 1B). Subsequently, we constructed *OsRbohH*-OE transgenic plants to investigate their functions further. The results showed that the *OsRbohH*-OE plants exhibited better growth after heat treatment, implying that *OsRbohH* is a positive regulator of heat tolerance (Figure 2A). Additionally, our results also indicate that overexpression of *OsRbohH* significantly improves drought resistance in rice, which agrees with the functions of *OsRbohA* and *OsRbohB* in drought stress [39,40]. However, whether *OsRbohA* and *OsRbohB* have a similar role to *OsRbohH* in heat tolerance needs further investigation. Since roots are the first organ to sense drought stress, the high transcript level of *OsRbohH* detected in roots by qRT-PCR (Figure 1A), which is consistent with the RPKM values analysis (Table S1), further demonstrates that *OsRbohH* plays a crucial role in responding to drought stress.

The mechanism responsible for the *OsRbohH*-regulated stress response is partially explained by several physiological changes observed in *OsRbohH*-OE plants. Firstly, the reduction in the rate of water loss in detached leaves may be one of the factors contributing to the improved drought resistance of *OsRbohH*-OE plants (Figure 3D). It is well established that the behavior of stomata on plant leaves significantly impacts water loss rates under drought-stress conditions. For instance, the increased closure of stomata plays a crucial role in enhancing the drought resistance of *OsRbohB*-OE plants [40]. However, whether *OsRbohH* plays a role in regulating stomatal behavior under drought-stress conditions remains to be elucidated. Secondly, maintaining the chlorophyll content and minimizing its degradation during stress significantly enhances photosynthetic efficiency [47]. In our findings, the total chlorophyll content in the *OsRbohH*-OE lines was considerably higher than that in WT plants following high-temperature and drought stress, suggesting that the stress-triggered degradation of chlorophyll was significantly mitigated in the *OsRbohH*-OE plants. It may be that the overexpression of *OsRbohH* alleviates pigment photooxidation and chlorophyll degradation caused by oxidative stress. Our results partially support this idea, as less ROS accumulated in *OsRbohH*-OE leaves than in WT after high-temperature and drought treatments (Figure 4A,B). Thirdly, considering that the generation of apoplastic ROS mediated by NADPH oxidases is a central mechanism of the plant stress response [15,16,17]. ROS also acts as an essential signal in upregulating the activities of antioxidant enzymes [48]. Previously, *OsRbohA*-OE plants have been reported to improve drought resistance by maintaining a relatively increased antioxidative capacity [39]. The mutants *AtrbohB* and *AtrbohD* show poor thermotolerance due to a deficiency in H_2_O_2_ [30]. Here, we observed that the activities of CAT and POD in *OsRbohH*-OE plants were significantly higher than those in WT under stress conditions (Figure 4C,D). Therefore, we speculate that the overexpression of *OsRbohH* may provide moderate levels of intracellular and apoplastic ROS, which contribute to higher heat and drought stress tolerance by upregulating the activities of antioxidant enzymes. However, this conclusion needs to be further verified by *osrbohh* knockout plants.

ABA is widely considered one of the critical regulators of plant responses to heat and drought [7]. In this study, *OsRbohH* overexpression lines exhibited increased sensitivity to exogenous ABA during seed germination (Figure 5A), implying an elevated endogenous ABA level in *OsRbohH*-OE plants. As reported, the accumulation of endogenous ABA in the plant leads to the production of ROS, which in turn activates the plant’s antioxidant system [49]. For instance, *ZFP36* regulates ABA-induced antioxidant defense, thus enhancing rice’s water and oxidative stress resistance [50]. *GmFUS3* controls the plant stress response by regulating the expression of ROS-scavenging enzyme genes by activating ABA biosynthesis [51]. It is considered that the production of ROS induced by ABA is primarily achieved by inducing the expression of genes that encode NADPH oxidase in plants [52]. For instance, ABA can activate NADPH oxidase gene expression, such as *OsRbohA*, *OsRbohC*, *OsRbohF*, and *OsRbohG,* in rice, thereby promoting the generation of ROS in cells [53]. Accumulation of ABA elevated the expression levels of *RBOH1*, leading to elevated apoplastic H_2_O_2_ accumulation and increased activity of antioxidant enzymes, thereby enhancing tomato salinity tolerance [54]. Therefore, one possible hypothesis is that the overexpression of *OsRbohH* leads to an increase in endogenous ABA levels, which in turn promotes the production of OsRbohH-mediated ROS. These increased ROS further activate the plant’s antioxidant system, thereby enhancing the drought stress tolerance of the *OsRbohH*-OE plant to a certain extent. Moreover, regulating stomatal closure is essential in mitigating water loss during water-deficient circumstances. ABA is the primary phytohormone that initiates stomatal closure [7]. Our study found that the expression of ABA signaling (*OsDREB2A*, *OsLEA3*, *OsbZIP66*, and *OsbZIP72*) genes was significantly upregulated in *OsRbohH*-OE plants under drought stress [55,56,57,58]. Therefore, another possible hypothesis is that *OsRbohH* may regulate ABA signaling under drought conditions, enhancing drought tolerance via ABA-mediated stomatal closure in plants. Interestingly, after high-temperature treatment, the expression of these ABA-signaling genes was significantly downregulated in the overexpression plants compared to WT (Figure 6). This seems to contradict the concept of ABA enhancing stress response and improving abiotic stress resistance. Increasing evidence has shown that ABA-mediated stomatal closure and transpiration can be disadvantageous under high-temperature conditions [44]. In plants that receive sufficient water, transpiration is the most effective method for cooling the leaves. For instance, under heat stress conditions, the heat-tolerant tomato varieties LA1994 and LA2093 exhibit larger stomatal sizes and relatively lower leaf temperatures, which can be attributed to their enhanced cooling capacity [59]. Transcripts of the ABA catabolism-related *CYP707A* gene in soybean flowers increase under heat stress, suggesting that the breakdown of ABA may play a central role in cooling the flowers of heat-stressed plants [60]. Additionally, some studies have shown that plants have developed intricate mechanisms to ensure that physiological signals are regulated correctly and are not overamplified. For instance, *OsbZIP23* enhances drought tolerance by feedback-regulating ABA signaling through direct interaction with the *OsPP2C* gene [61]. It is a consideration that our rice plants are grown hydroponically in a growth medium and are supplemented with water daily during the heat treatment and recovery periods. Therefore, we further conjecture that *OsRbohH* may reduce the accumulation of ABA under high-temperature conditions through a negative feedback regulation mechanism, thereby enhancing heat tolerance via ABA-mediated stomatal opening in plants, particularly when heat dissipation is prioritized over water preservation. However, the exact molecular mechanism of the possible negative regulation of ABA signaling by *OsRbohH* requires further detailed investigation. Moreover, we also found that *OsRbohH* transcription was significantly induced by the SA and MeJA treatments. It has been reported that SA can mitigate oxidative stress and enhance plant resilience in response to higher temperatures [7]. Jasmonic acid (JA) is a vital plant hormone that regulates the adaptation of plants to drought stress [7]. Therefore, it is worth further investigating whether *OsRbohH* influences the tolerance of transgenic plants to high temperatures and drought stress by modulating SA and JA levels.

## 4. Materials and Methods

### 4.1. Plant Materials and Cultivation Conditions

The plant materials used in this work included *OsRbohH*-overexpressing (*OsRbohH*-OE) plants and the rice variety Nipponbare. Nipponbare (NPB) was utilized as the recipient of genetic transformation in this study. Rice seedlings were grown under a 14/10 h light/dark cycle.

### 4.2. Generation of Transgenic Plants

To generate *OsRbohH* overexpression transgenic lines, the CDS (coding sequence) of *OsRbohH* was amplified by PCR and cloned into the pGWB512 vector. Subsequently, the recombinant plasmids were transferred into plants via *Agrobacterium*-mediated transformation. Table S2 has a list of the primer sequences that were employed.

### 4.3. Expression Profile Analysis

Samples of plant tissues were collected at various developmental stages for gene expression analysis, encompassing leaves, stems, and roots at the seedling stage and leaves, stems, roots, sheaths, internodes, and panicles at the heading stage. The rice seedlings were subjected to different treatments with heat (42 °C), 20% PEG6000 (*w*/*v*), cold (4 °C), and MeJA (100 µM), SA (100 µM) or ABA (400 µM). Leaf tissues were harvested at 0, 1, 2, 4, 8, and 12 h after high-temperature, drought, and low-temperature treatments. Similarly, leaf tissues were collected at 0, 3, 6, 9, and 12 h after ABA, MeJA, and SA treatments. Each sample consisted of three independent plant materials. SRA databases were used to analyze RPKM values.

### 4.4. Assays of Heat and Drought Treatment

In the heat tolerance assay, seedlings (n = 24) were incubated in a growth chamber at 45 °C for 3 days and then transferred to 28 °C for recovery. Survival rates were assessed after 7 days of recovery.

Two-week-old seedlings of *OsRbohH*-OE and WT plants (n = 24) were cultivated in identical pots and subjected to drought treatment. Afterward, a 7-day regular watering recovery period was conducted. After rehydration, plants displaying green and healthy leaves were deemed survivors. Survival rates were assessed after 7 days of recovery. To measure water loss, rice seedlings were placed on paper at room temperature and weighed at various intervals. The calculation formula was as follows: $\frac{\left(m0-m1\right)}{m0}\times 100\%$ (m0: initial leaf weight; m1: the mass of the foliage at the designated moment).

### 4.5. Analysis of ABA Sensitivity Assays

To evaluate the sensitivity to ABA at the germination stage, seeds from *OsRbohH*-OE and WT plants were germinated on a medium with either 0 μM ABA or 5 μM ABA. The length of the shoots was then measured on the fifth day after germination.

### 4.6. Gene Expression Analysis

A total RNA purification kit (Cowin, China) was used to extract the total RNA. Reverse mRNA transcription into cDNA was performed using a 5×All-In-One RT Master Mix (ABmart, Shanghai, China). The qPCR Master Mix (Vazyme, Nanjing, China) was then used for RT-qPCR. *OsActin* was used as the internal control gene. The qPCR protocol included an initial phase of 10 min at 95 °C, followed by 40 cycles of 10 s at 95 °C, 30 s at 60 °C, and 15 s at 95 °C, with a final 60 °C step lasting 1 min.

### 4.7. Measurement of Chlorophyll Concentrations

To determine the total chlorophyll content in the leaves, approximately 0.05 g of leaf material was weighed and extracted using 4 mL of an extraction liquid composed of distilled water (ddH_2_O), acetone, and absolute ethanol in a ratio of 1:4.5:4.5 (*v*/*v*). The mixture was shaken overnight in darkness to ensure thorough extraction, then centrifuged at 13,400× *g* at 8 °C for 12 min. The absorbance of the supernatant was measured at 663 nm and 645 nm wavelengths.

### 4.8. Measurement of Malondialdehyde Content

The aerial portions of the treated rice seedlings were collected, pulverized into powder with liquid nitrogen, and combined with a 5% (*w*/*v*) TCA buffer. The mixtures were centrifuged at 13,400× *g*, and the supernatant was mixed with a TBA buffer. It was promptly cooled after allowing the solution to react at 100 °C for 12 min. Finally, the solution was centrifuged at 13,400× *g* for 15 min, and a spectrophotometer was used to measure the absorbance of the supernatant at A_532_ and A_600_.

### 4.9. Electrolyte Leakage Measurements in Leaves

Three flag leaves from three plants were cut into uniformly sized segments. These segments were soaked in a test tube containing 25 mL of ddH_2_O for 12 h and continuously shaken. Then, the initial conductivity (G1) was measured. Afterward, the test tube containing the leaf segments was placed in a metal bath at 100 °C for 12 min and cooled naturally to room temperature. The electrical conductivity (G2) was measured again. The calculation formula was as follows: $\frac{\left(G1-G0\right)}{\left(G2-G0\right)}\times 100\%$.

### 4.10. Measurements of Antioxidant Enzyme Activities

The leaves of two-week-old rice seedlings were ground and placed in a phosphate buffer. The mixture was then centrifuged at 4 °C at 13,400× *g* for 20 min, and the antioxidant enzyme activity was determined using the supernatant. CAT activity was determined by analyzing the interaction between H_2_O_2_ and ammonium molybdate to form a yellow complex and then measuring the optical density (OD) value at 405 nm. One unit of CAT activity was defined as the amount of enzyme that transforms 1 µmol H_2_O_2_ within 1 min.

The determination of POD activity utilized guaiacol as the substrate. The activity was evaluated by monitoring changes in absorbance at 470 nm, and the results were expressed in U g^−1^min^−1^ (1 U corresponds to a decrease in absorbance of 0.01 at 470 nm per minute).

### 4.11. Detection of O_2_^−^ and H_2_O_2_ In Situ

To detect O_2_^−^ in situ, the seedlings were submerged in NBT solution at 27 °C for one day and then boiled in 95% ethanol to eliminate chlorophyll. The higher accumulation of dark blue-colored pigment indicated a higher level of O_2_^−^ in the rice.

To detect H_2_O_2_ in situ, the seedlings were submerged in DAB solution at 27 °C for one day and then boiled in 95% ethanol to eliminate chlorophyll. The levels of H_2_O_2_ were assessed through the intensity of dark brown-colored spots in rice.

### 4.12. Statistical Analysis

The data for each bar chart are presented as the mean  ±  SD. The Student’s *t*-test evaluated significant differences between the two samples (** *p* ≤ 0.01, * *p* ≤ 0.05).

## 5. Conclusions

This study found that *OsRbohH* was highly expressed in the roots and differentially regulated by abiotic stress and phytohormones. We identified *OsRbohH* as a positive regulator in response to drought and heat stress by generating *OsRbohH*-OE plants. These *OsRbohH*-OE plants showed increased survival rates, decreased ion leakage, and lower MDA content under heat and drought conditions, which could be partially attributed to the maintenance of ROS homeostasis. Additionally, *OsRbohH*-OE plants exhibited increased sensitivity to ABA during germination. Overexpression of *OsRbohH* also affected the transcript levels of ABA-responsive genes under drought and heat stress conditions, suggesting that the *OsRbohH* gene may modulate the ABA signaling pathway to enhance stress tolerance. However, the specific molecular mechanism by which *OsRbohH* functions under heat and drought stress needs to be further explored.

## Data Availability

All data from this study are available in this article and its Supplementary Materials.

## Supplementary Materials

The following supporting information can be downloaded at: https://www.mdpi.com/article/10.3390/plants13172494/s1, Figure S1: The expression levels of *OsRbohH* in three-week-old seedlings of *OsRbohH*-OE compared to those in WT. The expression level of *OsActin* was used as an internal control. The error bars indicate the ±SD based on three independent replicates. Asterisks indicate a significant difference determined by a two-tailed Student’s *t*-test at ***p*≤0.01. Figure S2: *OsRbohH* increases rice survival under drought-stress conditions. (A) The phenotype of *OsRbohH*-OE and WT plants grown in soil under normal and drought conditions. The 35-day-old WT and *OsRbohH*-OE plants were without water for 5 days and recovered under normal conditions for 7 days. (B) Survival rates of *OsRbohH*-OE and WT plants at 7 days after re-watering (n=3, with each replicate containing 42 plants). Data represent mean ± SD of three biological replicates from one experiment. Asterisks indicate a significant difference determined by a two-tailed Student’s *t*-test at ***p*≤0.01. Table S1. Tissue-specific expression profiles (in RPKM) for the *OsRbohH* gene. Table S2. Primer sequences used in this study.
