# Supplementary material for: Overexpression of OsRbohH Enhances Heat and Drought Tolerance through ROS Homeostasis and ABA Mediated Pathways in Rice (Oryza sativa L.)
Source: Plants (Basel). 2024 Sep 5;13(17):2494. doi: 10.3390/plants13172494 (PMC11397177; doi:10.3390/plants13172494)
Supplement: Supplementary file 1 [file plants-13-02494-s001.zip › plants-3078897-supplementary/Supplementary Files/Supplementary Figure.pdf]

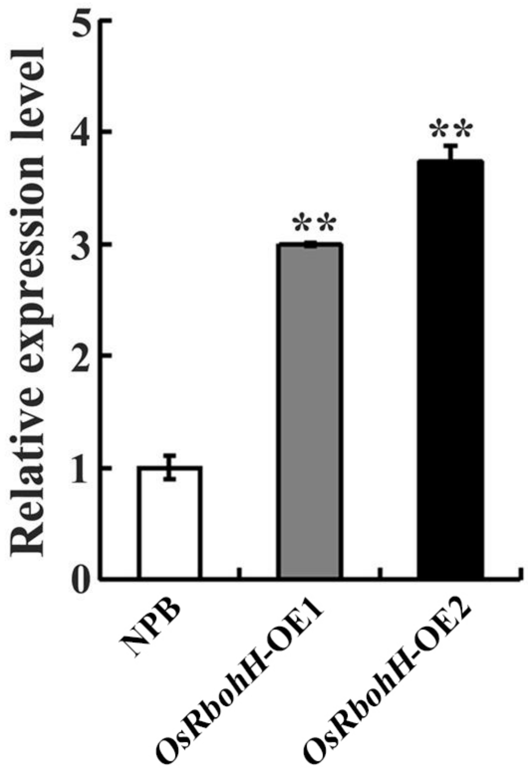

Figure S1: The expression levels of *OsRbohH* in three-week-old seedlings of *OsRbohH*-OE compared to those in WT. The expression level of *OsActin* was used as an internal control. The error bars indicate the ±SD based on three independent replicates. Asterisks indicate a significant difference determined by a two-tailed Student's *t*-test at $^{**}p \leq 0.01$.

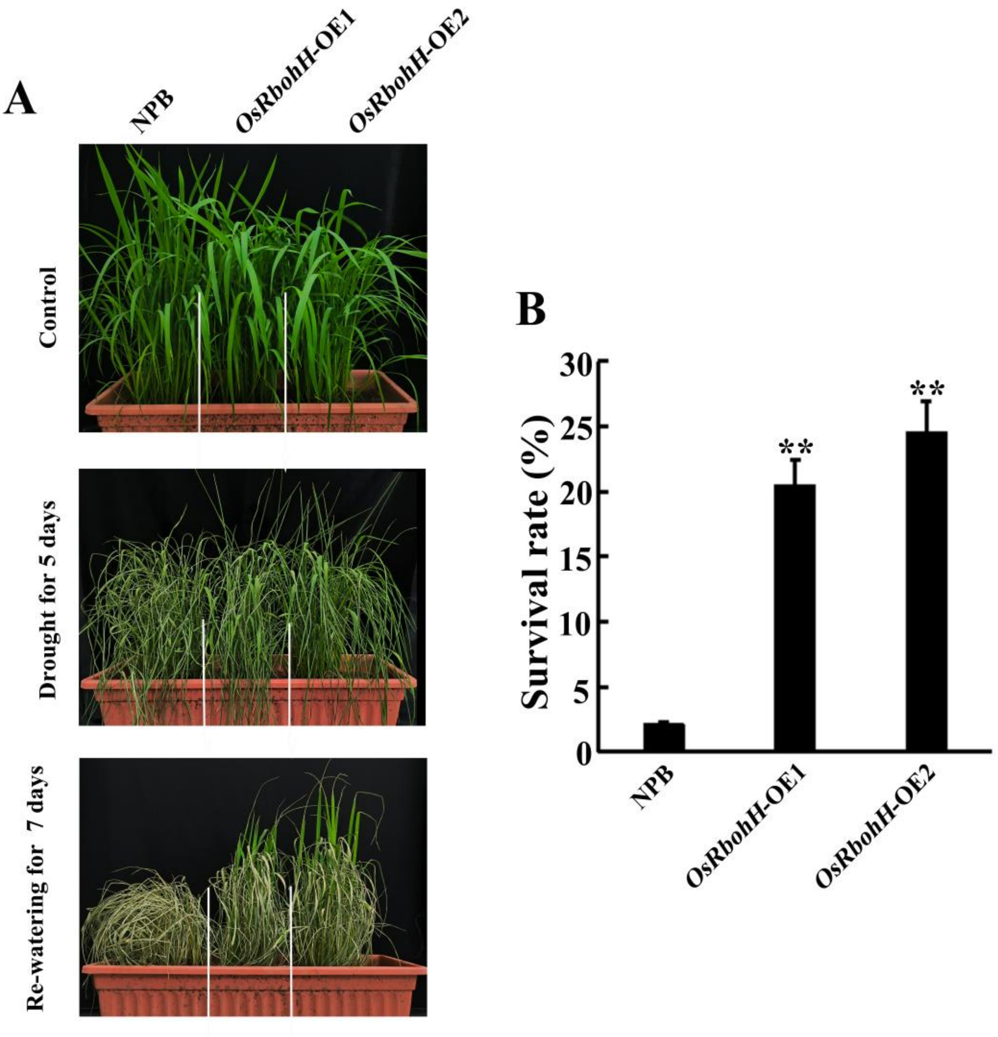

Figure S2: *OsRbohH* increases rice survival under drought-stress conditions. (A) The phenotype of *OsRbohH*-OE and WT plants grown in soil under normal and drought conditions. The 35-day-old WT and *OsRbohH*-OE plants were without water for 5 days and recovered under normal conditions for 7 days. (B) Survival rates of *OsRbohH*-OE and WT plants at 7 days after re-watering (n=3, with each replicate containing 42 plants). Data represent mean ± SD of three biological replicates from one experiment. Asterisks indicate a significant difference determined by a two-tailed Student's *t*-test at $^{**}p \leq 0.01$.
