# Supplementary material for: Overexpression of OsRbohH Enhances Heat and Drought Tolerance through ROS Homeostasis and ABA Mediated Pathways in Rice (Oryza sativa L.)
Source: Plants (Basel). 2024 Sep 5;13(17):2494. doi: 10.3390/plants13172494 (PMC11397177; doi:10.3390/plants13172494)
Supplement: Supplementary file 1 [file plants-13-02494-s001.zip › plants-3078897-supplementary/Supplementary Files/Table S1-2.pdf]

Table S1 Tissue-specific expression profiles (in RPKM) for the *OsRbohH* gene

Sample	RPKM	Count
Milk grains	4.12	113232
Mature seeds	3.026	52978
Roots	1.189	15283
Flowers	0.633	9771
Flower buds	0.475	6280
Leaves	0	0

Note: We used the dataset of RNA-Seq data from the SRA database. RPKM (gene expression in reads per exon kilobase per million mapped sequence reads); Count (Raw read count values).

Table S2 Primer sequences used in this study

Gene name	Forward primer (5'-3')	Reverse primer (5'-3')
OsRbohH (Os12g0541300)	TACTTCGGGCAGACACGGAT	GCGGGTTGCTGTCACTAAG
OsNCED3 (Os03g0645900)	AGTACTTCTACTTCGCGCCC	CGGGTACCACCACGTAGTTC
OsNCED4 (Os07g0154100)	GAGGTACGACTTCCATGGGC	TTGAGGTACGGCTTGGACAC
OsABA8ox3 (Os09g0457100)	CTCATCCACCACCTGGTCAC	ATCACCGTTCTGGCAACCAT
OsDREB2A (Os01g0165000)	TCCTTTCATCGTGGCTAA	TATTCTTCCGCTCCTGAC
OsLEA3 (Os05g0542500)	TTCCCACCAGGACCAGGCTA	GTCGCCTCCTTGGTATCCT
OsbZIP66 (Os08g0472000)	GGGGAATAGGCTGATGTCCG	GCTGCTGACTCCCTGTTCTT
OsbZIP72 (Os09g0456200)	GCGCAAGCAGGCTTACACATTG	GCCTGTTTCCTCTCCAATTCCTTG
OsActin (Os03g0718100)	GGCATTGCTGACAGGATGAG	GCTTAGCATTCTTGGGTCCG
OsRbohH-CDS (Os12g0541300)	GCAGGCTCAGGGGATATGGCG AGCCGGGAGGAGAG	CAGGGCGATATCGATATCGAAATT CTCC TTGTGGAAGA
